# Association of Endocrine Therapy Initiation Timeliness With Adherence and Continuation in Low-Income Women With Breast Cancer

**DOI:** 10.1001/jamanetworkopen.2022.25345

**Published:** 2022-08-03

**Authors:** Nikita Sood, Ying Liu, Min Lian, Tracy Greever-Rice, Jill Lucht, Chester Schmaltz, Graham A. Colditz

**Affiliations:** 1 Division of Public Health Sciences, Department of Surgery, Washington University School of Medicine in St Louis, Missouri; 2 Alvin J. Siteman Cancer Center at Barnes-Jewish Hospital, St Louis, Missouri; 3 Washington University School of Medicine in St Louis, Missouri; 4 Division of General Medical Sciences, Department of Medicine, Washington University School of Medicine in St Louis, Missouri; 5 Center for Health Policy, University of Missouri, Columbia, Missouri; 6 Department of Health Management and Informatics, University of Missouri School of Medicine, Columbia, Missouri

## Abstract

**Importance:**

Though adjuvant endocrine therapy (AET) has proven efficacy in treating hormone receptor–positive (HR-positive) breast cancer, patient adherence to AET and continuation of treatment as recommended by guidelines remain suboptimal, especially for low-income patients.

**Objective:**

To quantify timelines for initiating AET and assess their association with short- and long-term adherence and continuation of AET in low-income women with breast cancer.

**Design, Setting, and Participants:**

This population-based retrospective cohort study included women younger than 65 years diagnosed with first primary HR-positive breast cancer between January 1, 2007, and December 31, 2013, followed up for 5 years after the first use of AET through December 2018, and identified from the linked Missouri Cancer Registry and Medicaid claims data set.

**Exposures:**

Time to initiation (TTI) as days from the date of last treatment (surgery, radiotherapy, or chemotherapy) to the first date of AET prescription fill.

**Main Outcomes and Measures:**

The main outcomes were adherence to AET as medication possession ratio of 80% or greater and continuation of AET as no gap in medication supply for at least 90 days. Odds ratios (ORs) of adherence and continuation over 1 to 5 years were estimated using logistic regression adjusted for demographic, clinical, and neighborhood variables. Analyses were performed between September 1, 2020, and May 31, 2022.

**Results:**

Among 1711 patients, median TTI was 53 (IQR, 26-117) days. A total of 1029 patients (60.1%) were aged 50 to 64 years old, 1270 (74.2%) were non-Hispanic White, and 1133 (66.2%) were unmarried. In the first year after initiation, 1317 (77.0%) were adherent and 1015 (59.3%) continued AET. Over the full 5 years, 376 (22.0%) were adherent and 409 (23.9%) continued AET. Longer TTI was significantly associated with poorer adherence at every year, with an OR of 0.97 (95% CI, 0.95-0.99) for 1-year adherence and an OR of 0.94 (95% CI, 0.90-0.97) for 5-year adherence per 1-month increase in TTI. Longer TTI was also associated with lower odds of short-term, but not long-term, continuation (OR, 0.97 [95% CI, 0.95-0.99] for 1-year continuation and 0.98 [95% CI, 0.96-0.99] for 2-year continuation).

**Conclusions and Relevance:**

In this cohort study, longer time to AET initiation was associated with lower odds of short-term and long-term adherence to AET in Medicaid-insured patients with breast cancer. Therefore, early interventions targeting treatment initiation timelines may positively impact adherence throughout the course of treatment and, therefore, outcomes.

## Key Points

**Question:**

How are adjuvant endocrine therapy initiation timelines associated with adherence and continuation of treatment in low-income women with breast cancer?

**Findings:**

In this cohort study of 1711 women with breast cancer enrolled in Medicaid, longer time to initiation of adjuvant endocrine therapy was associated with lower odds of short-term and long-term adherence as well as short-term continuation.

**Meaning:**

These results suggest that interventions that focus on improving timeliness of treatment initiation may also improve adherence to treatment and therefore further improve outcomes, bringing benefits of advances to all women with breast cancer.

## Teaser

This cohort study examines the association between the timeliness of endocrine therapy initiation and treatment adherence and continuation among low-income women with breast cancer.

## Introduction

Breast cancer remains a prominent health concern for women. In the US, breast cancer is the most common type of cancer and the second leading cause of cancer death for women.^1^ While incidence rates of female breast cancer have remained relatively steady over the last 20 years, breast cancer mortality has been declining across all groups, in part because of advances in screening and treatment.^1,2,3,4^ However, breast cancer mortality remains pronounced among Black patients, who experience the highest rate compared to all other racial and ethnic groups, as well as among uninsured and Medicaid-insured patients and patients from counties with the highest poverty rates.^5,6,7^

Adjuvant endocrine therapy (AET) has contributed to the prognostic improvement for women with breast cancer, especially for hormone receptor–positive (HR-positive) subtypes, which make up approximately two-thirds of all breast cancer cases.^8^ Guidelines by the National Comprehensive Cancer Network and the American Society of Clinical Oncology underscore the importance of endocrine therapy as the primary systemic therapy for HR-positive breast cancers, recommending broadly that tamoxifen or aromatase inhibitor medications be taken daily for at least 5 years, with recent studies recommending as many as 10 years.^9,10,11^

Despite the proven efficacy of AET treatment,^12,13,14^ initiation, adherence, and continuation remain low and uneven among women. In Medicare-insured women with breast cancer, only 75% initiated AET within a year, and of those who initiated, approximately 75% were adherent for the first year.^15,16^ Lack of initiation is even more pronounced in low-income patients; only up to 60% of Medicaid enrollees with breast cancer initiated AET within a year, and of those who initiated, only 40% were adherent during the first year of treatment, with adherence further declining over time.^17,18,19^ Even fewer Medicaid-insured women continue AET for the recommended 5 years—as low as approximately 20% of patients.^18^ This nonadherence may lead to unequal distribution of the prognostic benefits of AET.

Although prior studies examined nonadherence and discontinuation with AET for women with HR-positive breast cancer and their associations with demographic variables,^15,18,20,21^ no study has examined whether initiation timing is associated with subsequent adherence to and/or continuation of AET. Therefore, we quantified timelines of AET initiation and their associations with short-term and long-term quality of AET in a population-based cohort of Medicaid-insured women with breast cancer.

## Methods

### Data Sources

For this population-based cohort study, we used the Medicaid administrative claims data to identify women who were enrolled in the fee-for-service program and diagnosed with breast cancer in Missouri. For the identified patients, their Medicaid claims for emergency, inpatient, and outpatient services and prescription drugs were linked to Missouri Cancer Registry data using Link Plus, version 2.0 (Centers for Disease Control and Prevention). Following the data collection and coding guidelines developed by the National Program of Cancer Registries, the Missouri Cancer Registry collects the diagnostic (date of diagnosis, primary site, cancer stage, tumor grade, tumor size, and HR status), treatment (dates of definitive surgery, first radiotherapy, first chemotherapy, and first hormone therapy prescription), and demographic (race, ethnicity, age, marital status, sex, and census tract) information for more than 95% of all incident cases of cancer in Missouri. A probabilistic matching approach was used, and matching variables included unique departmental control numbers, first and last name, social security number, date of birth, and race and ethnicity.^22,23^ The study was approved by the institutional review board at Washington University School of Medicine in St. Louis, the Missouri Department of Health and Senior Services, and the Missouri Department of Social Services with a waiver of consent granted for the use of deidentified data. We followed the Strengthening the Reporting of Observational Studies in Epidemiology (STROBE) reporting guideline.^24^

### Study Population

We identified women younger than 65 years who were diagnosed with first primary HR-positive (estrogen receptor and/or progesterone receptor positive) breast cancer between January 1, 2007, and December 31, 2013, and followed up for 5 years after the first use of AET (tamoxifen or aromatase inhibitors) through December 2018 (n = 2366). Patients were considered as continuously enrolled in Medicaid if they had fewer than 60 consecutive days of nonenrollment status per enrollment year. We excluded women who died or had subsequent breast cancers within 5 years after AET initiation (n = 460) and those who were not continuously enrolled in Medicaid for at least 5 years after AET initiation (n = 195). Our final sample consisted of 1711 women.

### Medication Variables

Time to initiation (TTI) was defined as the number of days from the date of last treatment (surgery, radiotherapy, or chemotherapy) to the first date of AET prescription fill. If AET was used simultaneously with other adjuvant therapy, TTI was 0. If endocrine therapy was used as neoadjuvant treatment, TTI was defined as the number of days from diagnosis to the first date of prescription fill. Claims data on national drug codes, dates of service, and days’ supply of medication were used to calculate the number of pills supplied for each period following AET initiation. Medication possession ratio—defined as the percentage of days in a given time period for which a patient had medication supply based on prescription fills—was used to estimate adherence to AET, with a ratio of 80% or greater considered adherent.^15,17,18,25^ Continuation was defined as having no gap in medication supply for at least 90 days during the relevant period.^17^ Adherence and continuation were evaluated for 1, 2, 3, 4, and 5 years following AET initiation.

### Covariates

Demographic variables including race and ethnicity (non-Hispanic Black, non-Hispanic White, or other), age (21-49 and 50-65 years), and marital status (married, unmarried, unknown) were obtained from the Missouri Cancer Registry. Race and ethnicity were included as variables because racial differences in adherence to AET have been documented. Neighborhood socioeconomic deprivation and rurality were assessed based on the census tracts of patients’ residence at diagnosis. The composite socioeconomic deprivation indices were calculated based on 21 variables from the 2005 to 2009 (for cases with 2000 residential census tract code available) and 2008 to 2012 (for cases with 2010 residential census tract code available) American Community Surveys, as described previously.^26,27,28^ Based on their statewide distribution, index scores were divided into quartiles, with a higher quartile suggesting greater socioeconomic deprivation. Rural census tracts were defined as nonmetropolitan areas and determined using the rural-urban commuting area codes from the US Department of Agriculture.^28^ Cancer stage was coded as 0 to IV based on the eighth edition of the *American Joint Committee on Cancer Staging Atlas*. The comorbidity index was computed using the algorithm developed by the National Cancer Institute and categorized as 0, 1, and 2 or greater.^29^

### Statistical Analysis

We used logistic regression to estimate the odds ratios (ORs) of adherence and continuation of AET from 1 to 5 years per monthly increase in TTI in the total sample (n = 1711). The ORs were adjusted for age, race and ethnicity, marital status, neighborhood socioeconomic deprivation, urban-rural residence, cancer stage, and comorbidities. The associations of these factors with adherence and continuation of AET were evaluated in cases with complete information (n = 1605) using multivariable logistic regression. All analyses were performed in SAS, version 9.4 (SAS Institute Inc). Statistical tests were all 2-sided, with statistical significance determined by *P* < .05. Analyses were performed between September 1, 2020, and May 31, 2022.

## Results

Among 1711 Medicaid-insured women with HR-positive breast cancer, 1029 (60.1%) were aged 50 to 64 years. A total of 404 women (23.6%) were non-Hispanic Black, 1270 (74.2%) were non-Hispanic White, and 37 (2.2%) were of other race or ethnicity (including Asian, Hispanic, American Indian or Alaska Native, or other); 1133 (66.2%) were unmarried; 594 (34.7%) were from areas in the highest quartile of socioeconomic deprivation; and 1196 (69.9%) were from urban areas (Table 1). Approximately 20% of patients had stage III (n = 266 [15.6%]) or IV (n = 59 [3.5%]) tumors. Multiple comorbid diseases were noted in 251 patients (14.7%). Overall median TTI was 53 days (IQR, 26-117 days).

**Table 1.** Characteristics of Medicaid-Insured Women With Hormone Receptor–Positive Breast Cancer Diagnosed Before 65 Years of Age in Missouri, 2007-2013

Characteristic	Overall, No. (%)	TTI, median (IQR), d^a^
Total	1711 (100)	53 (26-117)
Age at diagnosis, y		
21-49	682 (39.9)	55 (24-123)
50-64	1029 (60.1)	53 (27-113)
Race and ethnicity		
Non-Hispanic		
Black	404 (23.6)	53 (27-112)
White	1270 (74.2)	54 (25-117)
Other^b^	37 (2.2)	49 (23-124)
Marital status		
Married	552 (32.3)	50 (24-103)
Unmarried	1133 (66.2)	55 (27-124)
Unknown	26 (1.5)	61 (23-113)
Socioeconomic deprivation, quartile		
1st (lowest)	212 (12.4)	57 (28-113)
2nd	354 (20.7)	52 (23-112)
3rd	528 (30.9)	53 (26-105)
4th (highest)	594 (34.7)	52 (27-128)
Unknown	23 (1.3)	49 (15-112)
Urban-rural residency		
Urban	1196 (69.9)	54 (27-118)
Rural	481 (28.1)	52 (23-115)
Unknown	34 (2.0)	44 (15-91)
Cancer stage		
0	176 (10.3)	46 (24-99)
I	556 (32.5)	53 (27-99)
II	646 (37.8)	54 (26-120)
III	266 (15.6)	56 (26-135)
IV	59 (3.5)	89 (14-161)
Unknown	8 (0.5)	80 (54-218)
NCI Comorbidity index		
0	1036 (60.6)	53 (25-118)
1	424 (24.8)	53 (24-106)
≥2	251 (14.7)	56 (29-119

Abbreviations: NCI, National Cancer Institute; TTI, time to endocrine therapy initiation.

^a^ TTI was the number of days from the date of last treatment (surgery, radiotherapy, or chemotherapy) to the first date of endocrine therapy prescription fill. If endocrine therapy was used simultaneously with other adjuvant therapy, TTI was 0. If endocrine therapy was used as neoadjuvant treatment, TTI was the number of days from diagnosis to the first date of prescription fill.

^b^ The group included 13 Hispanic women, 16 Asian women, 2 American Indian or Alaska Native women, and 6 women of other races.

**Figure 1.**
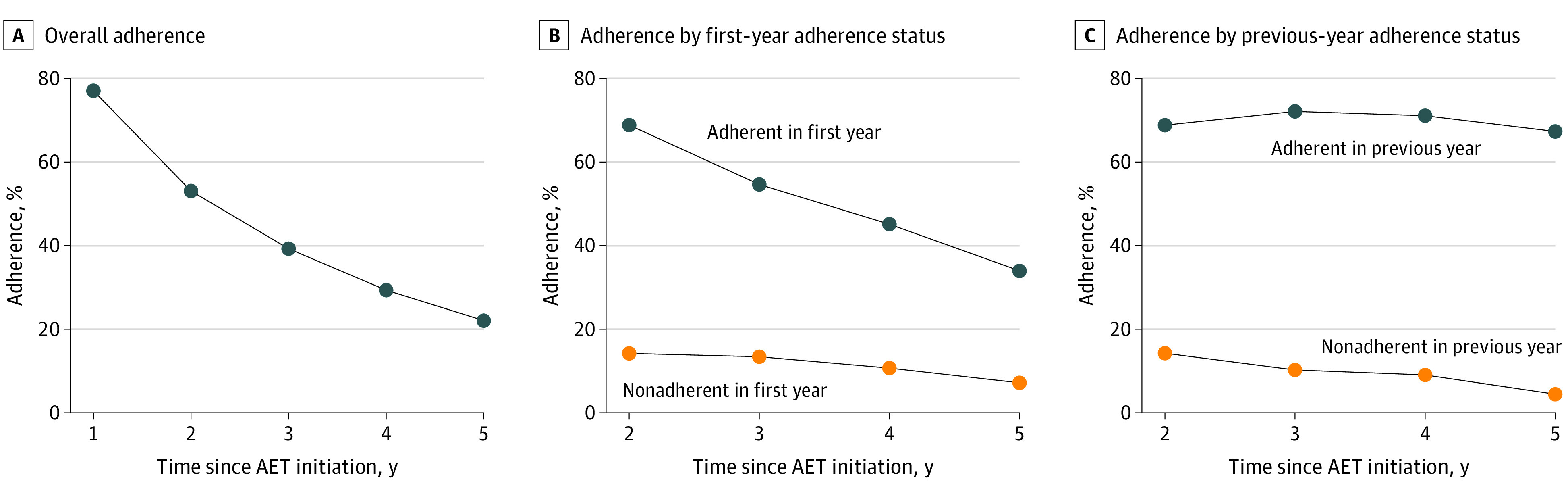
Adherence to Endocrine Therapy Over Time in Medicaid-Insured Women With Hormone Receptor–Positive Breast Cancer Adherence was defined using a medication possession ratio (percentage of days covered by medication supply in a specified period) of at least 80%. A, Adherence over years after endocrine therapy initiation. B, Adherence in each subsequent year by adherence status in the first year of endocrine therapy. C, Adherence in each subsequent year by adherence status in the previous year of endocrine therapy. AET indicates adjuvant endocrine therapy.

**Figure 2.**
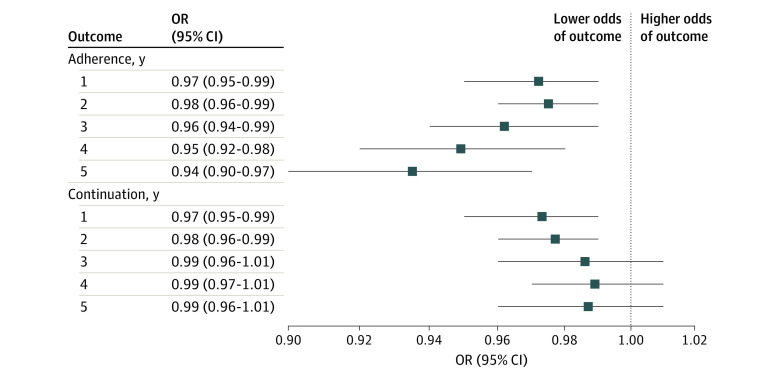
Associations of Time to Endocrine Therapy With Treatment Adherence and Continuation in Patients With Breast Cancer The odds ratio (OR) was for a month increase in time to initiation of endocrine therapy and was adjusted for age, race and ethnicity, marital status, census tract–level socioeconomic deprivation, rural residency, cancer stage, and comorbidity index.

Adherence to AET decreased monotonically over time, from 1317 patients (77.0%) adherent for the first year to 376 (22.0%) adherent for the full 5 years (Figure 1A). Compared with patients who were not adherent in year 1, a larger proportion of patients who were adherent in the first year maintained adherence for later years, with 906 patients (68.8%) (vs 56 [14.2%]) still adherent in year 2 and 447 patients (33.9%) (vs 28 [7.1%]) still adherent in year 5 (Figure 1B). Regarding incremental trends, patients who were adherent in the previous year were more likely to maintain adherence for the subsequent year compared with those who were not adherent in the year prior (Figure 1C). Longer TTI was significantly associated with lower likelihood of adherence, with an OR of 0.97 (95% CI, 0.95-0.99) in the first year vs an OR of 0.94 (95% CI, 0.90-0.97) in a full 5-year duration for 1-month increases in TTI (Figure 2).

We also assessed the associations between demographic and clinical factors and adherence to AET (Table 2). Rural patients had significantly higher odds of adherence for 2 years (OR, 1.43; 95% CI, 1.12-1.82) to 5 years (OR, 1.65; 95% CI, 1.24-2.20) compared with urban patients. The likelihoods of adherence for the first year (OR, 0.63; 95% CI, 0.48-0.84), and 5 years (OR, 0.73; 95% CI, 0.56-0.94) were significantly lower in unmarried compared with married women. Women with multiple comorbidities had a significantly and consistently lower odds of adherence for 1 year (OR, 0.46; 95% CI, 0.33-0.64) to 5 years (OR, 0.37; 95% CI, 0.24-0.58) compared with women with no comorbidity. Women with stage IV tumors were less likely than women with stage I tumors to adhere to treatment for 4 years (OR, 0.37; 95% CI, 0.17-0.81) and 5 years (OR, 0.32; 95% CI, 0.12-0.83). In the first year, significantly lower odds of adherence were observed in non-Hispanic Black patients (OR, 0.62; 95% CI, 0.46-0.83) and patients living in the most deprived (highest quartile) neighborhoods (OR, 0.65; 95% CI, 0.42-0.99), and significantly higher odds were seen in patients with stage 0 tumors (OR, 2.10; 95% CI, 1.31-3.37) compared to their counterparts; however, no significant difference in odds of adherence was observed at any other time. Age was not associated with odds of adherence.

**Table 2.** Odds Ratios of Adherence and Continuation of Adjuvant Endocrine Therapy Over Time Associated With Demographic Factors in Medicaid-Insured Women With Hormone Receptor–Positive Breast Cancer Diagnosed Before 65 Years of Age

	1 y		2 y			3 y		4 y		5 y	
	Adherent/continued, %	OR (95% CI)^a^	Adherent/continued, %	OR (95% CI)^a^	Adherent/continued, %		OR (95% CI)^a^	Adherent/continued, %	OR (95% CI)^a^	Adherent/continued, %	OR (95% CI)^a^
**Adherence^b^**											
Age, y											
21-49	77.4	1 [Reference]	51.6	1 [Reference]	36.6		1 [Reference]	28.0	1 [Reference]	20.6	1 [Reference]
50-64	76.1	1.05 (0.82-1.35)	52.8	1.11 (0.90-1.37)	40.5		1.27 (1.00-1.57)	29.6	1.14 (0.90-1.43)	22.6	1.23 (0.95-1.59)
Race and ethnicity											
Non-Hispanic Black	68.4	0.62 (0.46-0.83)	47.2	0.87 (0.68-1.12)	35.5		0.97 (0.75-1.27)	25.3	0.98 (0.73-1.31)	18.1	0.95 (0.68-1.31)
Non-Hispanic White	79.3	1 [Reference]	53.9	1 [Reference]	40.1		1 [Reference]	30.2	1 [Reference]	23.0	1 [Reference]
Marital status											
Married	83.6	1 [Reference]	55.7	1 [Reference]	42.4		1 [Reference]	33.3	1 [Reference]	27.2	1 [Reference]
Unmarried	73.3	0.63 (0.48-0.84)	50.6	0.91 (0.73-1.13)	37.3		0.90 (0.72-1.12)	26.9	0.81 (0.64-1.03)	19.2	0.73 (0.56-0.94)
Socioeconomic deprivation, quartile											
1st (least deprived)	82.4	1 [Reference]	51.3	1 [Reference]	37.7		1 [Reference]	29.2	1 [Reference]	23.1	1 [Reference]
2nd	77.5	0.69 (0.44-1.09)	53.5	1.04 (0.73-1.48)	38.3		0.98 (0.68-1.41)	28.4	0.91 (0.61-1.35)	19.0	0.74 (0.47-1.14)
3rd	75.6	0.58 (0.38-0.90)	52.9	0.94 (0.67-1.32)	42.1		1.05 (0.74-1.50)	30.9	0.92 (0.63-1.34)	23.3	0.83 (0.55-1.26)
4th (most deprived)	75.0	0.65 (0.42-0.99)	51.3	0.92 (0.65-1.30)	37.0		0.87 (0.61-1.25)	27.6	0.82 (0.56-1.21)	21.8	0.82 (0.54-1.25)
Urban-rural residency											
Urban	75.2	1 [Reference]	49.6	1 [Reference]	36.5		1 [Reference]	26.3	1 [Reference]	19.2	1 [Reference]
Rural	80.4	1.24 (0.92-1.67)	59.0	1.43 (1.12-1.82)	45.2		1.43 (1.12-1.83)	35.7	1.56 (1.20-2.03)	28.4	1.65 (1.24-2.20)
Cancer stage											
0	83.6	2.10 (1.31-3.37)	52.1	1.01 (0.71-1.44)	38.8		1.03 (0.71-1.49)	27.3	0.86 (0.58-1.29)	19.4	0.83 (0.53-1.30)
I	73.6	1 [Reference]	52.9	1 [Reference]	39.0		1 [Reference]	31.1	1 [Reference]	23.2	1 [Reference]
II	79.0	1.31 (0.98-1.74)	51.9	0.96 (0.76-1.22)	41.0		1.11 (0.87-1.42)	29.6	0.94 (0.72-1.21)	22.8	0.99 (0.74-1.32)
III	73.9	0.96 (0.67-1.37)	53.0	0.99 (0.73-1.34)	36.6		0.90 (0.65-1.23)	27.3	0.82 (0.58-1.15)	20.9	0.85 (0.58-1.24)
IV	70.9	0.85 (0.45-1.59)	47.3	0.79 (0.45-1.38)	27.3		0.59 (0.32-1.11)	14.6	0.37 (0.17-0.81)	9.1	0.32 (0.12-0.83)
NCI Comorbidity index											
0	79.4	1 [Reference]	54.2	1 [Reference]	40.9		1 [Reference]	30.7	1 [Reference]	24.2	1 [Reference]
1	77.4	0.87 (0.65-1.16)	52.9	0.91 (0.72-1.16)	39.6		0.89 (0.70-1.14)	30.3	0.93 (0.72-1.21)	22.1	0.83 (0.62-1.11)
≥2	64.0	0.46 (0.33-0.64)	43.2	0.61 (0.46-0.83)	29.7		0.56 (0.41-0.77)	19.5	0.51 (0.36-0.74)	11.4	0.37 (0.24-0.58)
**Continuation^c^**											
Age, y											
21-49	58.4	1 [Reference]	41.0	1 [Reference]	30.2		1 [Reference]	25.6	1 [Reference]	23.2	1 [Reference]
50-64	60.2	1.05 (0.85-1.30)	41.4	1.03 (0.83-1.27)	31.3		1.08 (0.86-1.35)	26.4	1.10 (0.87-1.40)	24.2	1.13 (0.89-1.45)
Race											
Non-Hispanic Black	52.8	0.75 (0.58-0.97)	30.6	0.55 (0.42-0.71)	22.7		0.58 (0.44-0.78)	19.9	0.61 (0.45-0.83)	18.1	0.63 (0.46-0.87)
Non-Hispanic White	61.7	1 [Reference]	44.7	1 [Reference]	33.5		1 [Reference]	28.0	1 [Reference]	25.6	1 [Reference]
Marital status											
Married	64.4	1 [Reference]	45.5	1 [Reference]	33.9		1 [Reference]	28.1	1 [Reference]	26.4	1 [Reference]
Unmarried	57.2	0.78 (0.63-0.98)	39.2	0.86 (0.69-1.07)	29.4		0.89 (0.71-1.13)	25.1	0.93 (0.73-1.19)	22.6	0.89 (0.69-1.14)
Socioeconomic deprivation. quartile											
1st (least deprived)	52.3	1 [Reference]	40.7	1 [Reference]	33.2		1 [Reference]	31.2	1 [Reference]	30.2	1 [Reference]
2nd	62.3	1.47 (1.03-2.11)	43.3	1.11 (0.77-1.59)	33.9		1.03 (0.71-1.50)	29.5	0.94 (0.64-1.38)	27.8	0.90 (0.61-1.33)
3rd	61.1	1.35 (0.96-1.90)	41.5	1.01 (0.71-1.43)	30.7		0.88 (0.61-1.27)	25.1	0.76 (0.52-1.11)	22.7	0.69 (0.47-1.01)
4th (most deprived)	58.9	1.32 (0.93-1.86)	40.0	1.06 (0.75-1.51)	28.3		0.87 (0.60-1.26)	23.0	0.74 (0.50-1.08)	20.2	0.64 (0.43-0.94)
Urban-rural residency											
Urban	58.0	1 [Reference]	40.1	1 [Reference]	30.4		1 [Reference]	26.5	1 [Reference]	24.2	1 [Reference]
Rural	63.4	1.06 (0.83-1.36)	44.1	0.98 (0.77-1.25)	31.9		0.96 (0.74-1.25)	24.9	0.87 (0.66-1.15)	22.9	0.92 (0.70-1.23)
Cancer stage											
0	58.8	0.99 (0.69-1.42)	43.0	1.25 (0.87-1.80)	34.6		1.31 (0.90-1.91)	29.7	1.46 (0.98-2.16)	29.1	1.60 (1.07-2.39)
I	60.3	1 [Reference]	40.1	1 [Reference]	30.7		1 [Reference]	24.1	1 [Reference]	22.0	1 [Reference]
II	58.8	0.94 (0.74-1.20)	42.0	1.08 (0.85-1.38)	29.9		0.97 (0.75-1.25)	26.1	1.12 (0.86-1.48)	24.0	1.13 (0.85-1.49)
III	58.6	0.95 (0.69-1.30)	41.0	1.06 (0.78-1.45)	31.3		1.05 (0.75-1.46)	27.7	1.22 (0.86-1.73)	23.7	1.10 (0.76-1.58)
IV	65.5	1.24 (0.69-2.23)	40.0	1.01 (0.57-1.79)	29.1		0.96 (0.52-1.77)	25.5	1.14 (0.60-2.17)	23.6	1.17 (0.60-2.27)
NCI Comorbidity index											
0	58.5	1 [Reference]	41.7	1 [Reference]	31.9		1 [Reference]	27.5	1 [Reference]	25.8	1 [Reference]
1	61.2	1.08 (0.84-1.37)	39.4	0.88 (0.69-1.12)	29.6		0.88 (0.68-1.14)	22.8	0.77 (0.58-1.02)	20.1	0.71 (0.53-1.00)
≥2	61.0	1.10 (0.81-1.49)	42.8	1.05 (0.78-1.41)	28.8		0.86 (0.62-1.18)	25.4	0.90 (0.65-1.26)	22.0	0.82 (0.58-1.16)

Abbreviations: NCI, National Cancer Institute; OR, odds ratio.

^a^ All factors in the table and time to endocrine therapy initiation were simultaneously included in the models.

^b^ Adherence was defined using a medication possession ratio (percentage of days covered by medication supply in a specified period) of at least 80%.

^c^ Continuation was defined as having no gap in medication supply for at least 90 days in a specified period.

**Figure 3.**
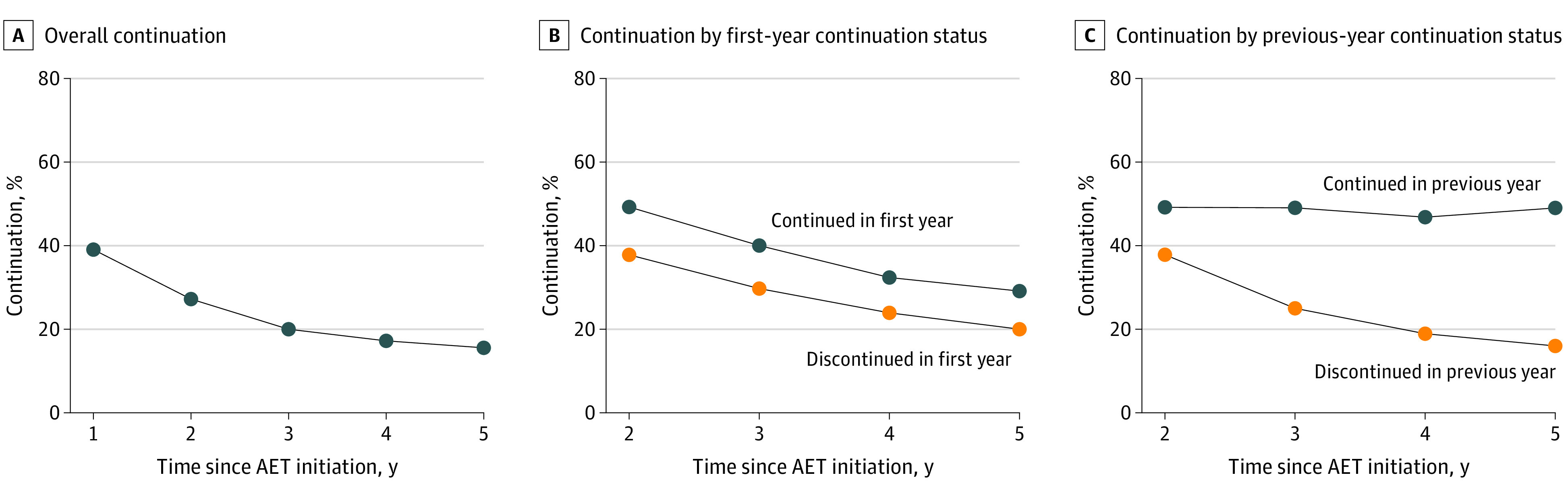
Continuation of Endocrine Therapy Over Time in Medicaid-Insured Women With Hormone Receptor–Positive Breast Cancer Continuation was defined as having no gap in medication supply for at least 90 days in a specified time period. A, Continuation over years after endocrine therapy initiation. B, Continuation in each subsequent year by continuation status in the first year of endocrine therapy. C, Continuation in each subsequent year by adherence status in the previous year of endocrine therapy. AET indicates adjuvant endocrine therapy.

Continuation of AET showed similar trends over time to adherence, with 1015 patients (59.3%) persisting for the first year and 409 (23.9%) persisting for 5 years (Figure 3A). Patients who continued AET in the first year were more likely to continue AET in later years compared with those who discontinued AET in year 1, with 499 patients (49.2%) (vs 263 [37.8%]) still persistent in year 2 and 295 patients (29.1%) (vs 140 [20.1%]) still persistent in year 5 (Figure 3B). When evaluated incrementally, year-to-year continuation was relatively stable, with 46.8% to 49.2% of the patients who continued in the previous year continuing treatment in the subsequent year. However, incremental continuation for patients who did not continue in the year prior declined over time, with 263 patients (37.8%) who discontinued in year 1 continuing through year 2 and 193 patients (15.9%) who discontinued in year 4 continuing through year 5 (Figure 3C). Longer TTI was significantly associated with lower likelihood of continuation over the first year (OR, 0.97; 95% CI, 0.95-0.99) and 2 years (OR, 0.98; 95% CI, 0.96-0.99) (Figure 2). This association was not observed for continuation for 3 or more years.

Race and ethnicity, marital status, socioeconomic deprivation, and cancer stage were significantly associated with odds of continuation (Table 2). Non-Hispanic Black patients had consistently lower odds of continuation over time compared with non-Hispanic White patients (for the first year: OR, 0.75; 95% CI, 0.58-0.97; for 5 years: OR, 0.63; 95% CI, 0.46-0.87). Unmarried patients had lower odds of 1-year continuation compared with married patients (OR, 0.78; 95% CI, 0.63-0.98). Patients residing in the most deprived neighborhoods had lower odds of 5-year continuation (OR, 0.64; 95% CI, 0.43-0.94), and patients with stage 0 tumors had higher odds of 5-year continuation (OR, 1.60; 95% CI, 1.07-2.39) compared to their counterparts. No significant associations were found for age, urban-rural residence, or comorbidities at any year.

## Discussion

In Medicaid-insured women with breast cancer, we observed that longer time to AET initiation was significantly and independently associated with lower odds of short-term and long-term adherence to AET, as well as lower odds of continuation in the first 2 years. These results suggest that more timely initiation of AET—perhaps even sooner than the typical 1-year threshold—is associated with better adherence and continuation of the treatment in years following. Adherence and continuation in the first year of treatment were associated with higher rates of adherence and continuation in years 2 through 5, suggesting the key role of the first year of treatment in developing optimal medication behavior patterns.

To our knowledge, no study has quantified AET initiation timelines and examined their association with adherence or continuation. Most existing research has treated initiation as occurring or not within 1 year of diagnosis.^15,18^ Research in other treatment populations has similarly found that delayed treatment initiation is associated with poorer adherence, possibly owing to the role of timely initiation in emphasizing the importance of treatment.^30^ This possibility aligns with research that has found perceived importance of AET to be a key factor in shaping adherence and continuation.^31,32^ Initiation timeliness may also highlight barriers to care that further affect adherence and continuation, such as burdensome copayments or logistical challenges with Medicaid enrollment. Although delaying initiation of AET has not been linked to worse outcomes—possibly because studies have not probed initiation timelines beyond 1 year—poorer adherence and continuation have.^13,33,34,35,36^ Our findings suggest that improvement in initiation timeliness—and, therefore, adherence and continuation—could serve as a key opportunity for interventions in improving outcomes.

As expected, patients who were adherent for a given year had higher rates of adherence in the year following compared with patients who had not been adherent; these rates of adherence based on behaviors in the previous year remained relatively constant over time. Continuation similarly showed that patients who continued for a given year had higher rates of continuation in the subsequent year compared with patients who had not continued. However, rates of continuation in the following year for patients who discontinued in the year prior worsened over time, suggesting that poor continuation habits early on had a greater chance of being corrected in the following year compared with poor continuation habits later in the 5-year period.

Our results for adherence and continuation contribute to a growing body of research reporting suboptimal rates among breast cancer patients, especially low-income populations. Only 77.0% of Medicaid-insured women in our study were adherent to AET for the first year, and only 22.0% were adherent for 5 years.^9,11^ Prior studies of adherence in Medicaid-insured women reported that 55% to 60% were adherent in the first year, compared with 84% of Medicare-insured women.^15,17,18,21,37,38,39^ These differences suggest a role for insurance status and/or incomes in adherence trends; these findings may also be attributed to age differences between the 2 populations, although this seems less likely given the strong association between socioeconomic status and medication costs and treatment adherence.^37,40^ Fewer studies have examined continuation of AET in any population of patients with breast cancer. Approximately 80% of Medicare-insured patients discontinued AET within the 4 years, and 80% of Medicaid-insured patients discontinued within 5 years.^18,41^ Studies have highlighted the complex relationship between demographic and clinical characteristics, patient perceptions, and logistical barriers in influencing adherence and continuation. For this population of Medicaid-insured women in Missouri, enrollment and approval procedures for treatment coverage, ability to pay copays ($0.50 to $10), and logistical barriers, such as access to transportation to acquire prescriptions, may also affect health behaviors.^23,42,43,44^ Further investigations into factors associated with AET adherence and continuation in Medicaid-insured patients are crucial for developing interventions to address these suboptimal trends.

This study demonstrated that non-Hispanic Black patients had lower odds of both adherence and continuation compared with non-Hispanic White patients, despite the average TTI for both groups being comparable. Population-based studies have reported significantly lower odds of adherence in non-Hispanic Black patients than in non-Hispanic White patients,^16,17,18,20,21,45^ although other population-based and hospital-based studies have not observed such an association.^15,32,46^ Associations between race and AET continuation are even less substantiated. Studies of Medicaid-insured women have found no racial difference in continuation,^18,20,21,32,46,47,48^ and others have found higher rates of continuation among Medicare-insured non-Hispanic Black women.^41^ Variations in findings may be attributed to population-level trends in type of AET used as well as population differences, such as insurance status, because studies in Medicaid-insured women tend to find lower odds of adherence among non-Hispanic Black compared with non-Hispanic White patients. Regardless, non-Hispanic Black patients continue to face systemic barriers to care and have suboptimal outcomes compared with their non-Hispanic White counterparts. Persistent racial disparities in breast cancer highlight the need for investigations of factors associated with nonadherence and discontinuation of AET in non-Hispanic Black women.

Regarding associations between adherence and continuation and other factors, married patients had higher odds of adherence and continuation compared with unmarried patients, consistent with the few studies that have examined these associations.^13,15,46,49^ Additionally, patients from areas of the least socioeconomic deprivation had higher odds of adherence and continuation compared with their counterparts, which is consistent with existing research in which patients from areas of lower socioeconomic status had lower rates of adherence and continuation.^16,50^ Together, these findings contribute to the idea that social support, as evidenced through interpersonal relationships and economic stability, can affect health behaviors. Regarding clinical factors, patients with higher comorbidity indices and stage IV cancer had lower odds of adherence, and patients with higher comorbidity indices also had lower odds of continuation compared with their counterparts; these findings with existing research suggesting that disease burden can negatively impact health behaviors.^32,43,51,52^

### Limitations

This research had limitations. Our estimates of TTI, adherence, and continuation were based on Medicaid claims, which may not reflect actual patient behaviors. Additionally, our study population only focused on patients in Missouri younger than 65 years who were enrolled in Medicaid for at least 5 years. Further research into other populations could determine the association of AET initiation timelines with adherence and continuation among patients older than 65 years and those who may not have access to consistent insurance coverage. Additional research into factors influencing initiation timelines, which were not covered in this study, could also clarify opportunities for intervention.

## Conclusions

The association between TTI and adherence as well as the downward trends in adherence and continuation found in this cohort study suggest that interventions focused on improving timeliness of AET initiation may also promote adherence to and continuation of treatment. This opportunity could further improve outcomes, bringing benefits of advances to all women with breast cancer. Our findings in incremental adherence and continuation suggest that focusing on the first year of treatment may be of most value. Given the lack of success with previous attempts to improve health behaviors,^53^ these findings provide a promising new avenue for intervention.
